# An Open Label, Cross-Over Phase 1 Study to Determine the Safety, Tolerability and Pharmacokinetics of Multiple Oral Doses of Niclosamide Under Fed and Fasted Conditions in Healthy Volunteers

**DOI:** 10.3390/jcm15145330

**Published:** 2026-07-08

**Authors:** Gary K. Ostrander, Eric H. Holmes

**Affiliations:** Department of Biomedical Sciences, College of Medicine, Florida State University, Tallahassee, FL 32306, USA

**Keywords:** Zika virus, Yomesan^®^, PK, drug repurposing, antiviral, oral bioavailability

## Abstract

**Background/Objectives:** Niclosamide is an U.S. Food and Drug Administration (FDA)-approved antihelminthic drug with a long-established safety profile and demonstrated in vitro antiviral activity against Zika virus. Its evaluation for systemic indications has been limited by its poor oral bioavailability. This Phase I study assessed whether oral administration of the approved niclosamide formulation could achieve plasma concentrations comparable to those with reported antiviral activity. **Methods:** This single-center, open-label, randomized, two-period crossover Phase I study evaluated the safety, tolerability, and pharmacokinetics of oral niclosamide in healthy adult volunteers. Twelve participants received niclosamide 2 g once daily for three consecutive days under fed or fasted conditions, followed by crossover after a 14-day washout period. Plasma niclosamide concentrations were quantified using a validated Liquid Chromatography-Mass Spectrometry (LC–MS/MS) assay, and pharmacokinetic parameters were derived via non-compartmental analysis. Safety assessments included adverse event monitoring, clinical laboratory testing, vital signs, electrocardiograms, and physical examinations. **Results:** Nine participants per protocol completed the study. Niclosamide was safe and well tolerated under both fed and fasted conditions, with only mild to moderate, transient adverse events and no serious or severe events. Systemic exposure was markedly higher following fed administration, with mean maximum observed plasma concentration (C_max)_ and Area Under the Curve (AUC) values several-fold greater than those observed under fasted conditions. Under fed conditions, mean plasma niclosamide concentrations exceeded the reported in vitro Zika virus half-maximal inhibitory concentration (IC_50)_ (0.22 µM) for approximately 9 h post-dose on both Day 1 and Day 3. Fasted administration did not consistently achieve this exposure threshold. No unexpected accumulation was observed with repeated once-daily dosing. **Conclusions:** Oral niclosamide administered at the approved daily dose of 2 g is safe and well tolerated in healthy volunteers. Administration with food substantially enhances systemic exposure and transiently achieves plasma concentrations associated with in vitro antiviral activity against Zika virus. These findings support further development of optimized niclosamide formulations to achieve sustained systemic exposure for antiviral therapeutic applications.

## 1. Introduction

Niclosamide (5-chloro-N-(2-chloro-4-nitrophenyl)-2-hydroxybenzamide) is a chlorinated salicylanilide compound originally developed in the 1950s by scientists at Bayer [1]. First synthesized in 1953, niclosamide was initially recognized for its molluscicidal properties and subsequently commercialized as Bayluscide^®^ in 1959 for the control of snail vectors responsible for transmission of schistosomiasis [2]. Shortly thereafter, its anthelmintic activity was demonstrated [3], leading to approval by the U.S. Food and Drug Administration in 1982 for the treatment of human tapeworm infections under the trade name Yomesan^®^ [4]. The therapeutic effect of niclosamide against helminths is primarily attributed to inhibition of mitochondrial oxidative phosphorylation, resulting in energy depletion and parasite death.

Niclosamide is classified as a Biopharmaceutics Classification System (BCS) Class IV compound, reflecting poor aqueous solubility and low intestinal permeability. It is nonpolar and demonstrates approximately 10% oral bioavailability in animal models [5]. These pharmacokinetic characteristics are advantageous for treatment of gastrointestinal helminth infections, as high concentrations are localized to the intestinal lumen, but its low oral bioavailability has limited the development of niclosamide for systemic therapeutic indications. Consequently, for many decades, niclosamide was not pursued beyond its established use as an anthelmintic. Nevertheless, the drug has a wide safety margin [2], with no evidence of teratogenicity in animal studies [1], and low acute toxicity, with an oral median lethal dose (LD_50_) of approximately 5000 mg/kg in rats [2].

In recent years, niclosamide has received renewed attention as a candidate for drug repurposing due to its broad pharmacological activity and favorable safety profile. In vitro studies have demonstrated potent inhibition of Zika virus replication, with a reported 50% inhibitory concentration of 0.22 μM [6]. Comparable concentrations have been associated with reduced cell viability and proliferation in human and canine osteosarcoma cell lines [7], as well as modulation of additional cellular signaling pathways implicated in oncogenesis and inflammation [8]. Although niclosamide remains included on the World Health Organization’s Model List of Essential Medicines, it is no longer marketed for human use in the United States, where current utilization is largely restricted to veterinary applications for the treatment of parasitic infections.

Preclinical and translational studies have evaluated niclosamide in a range of disease models, including pancreatic cancer [9], leukemia [10], colorectal cancer [11], multiple myeloma [12], renal ischemia–reperfusion injury [13], bacterial infection [14], endometriosis [15], and viral diseases [16]. In the context of Zika virus infection, niclosamide has been shown to inhibit replication of multiple viral strains and to suppress caspase-3-mediated cell death in infected glioblastoma cells, human neural progenitor cells, and astrocytes [17,18]. Additionally, niclosamide analogs with improved anti-Zika properties have been developed [6,19]. Despite these findings, no clinical studies have yet evaluated niclosamide for the treatment or prevention of Zika virus infection in humans.

The systemic exposure to niclosamide in humans following oral administration of the approved Yomesan^®^ product is unknown, although it is generally well tolerated. Reported adverse events are infrequent, typically mild and transient, and most commonly include gastrointestinal discomfort, nausea, vomiting, diarrhea, dizziness, headache, and pruritic skin rash. Serious adverse effects have not been reported in clinical use.

To determine the systemic exposure of niclosamide arising from the 2 g/day approved dose, we conducted a pharmacokinetic evaluation of oral niclosamide as a first step to guide future reformulation efforts for systemic antiviral therapy targeting Zika virus infection. Our objectives were to characterize plasma niclosamide concentrations under fed and fasted conditions following administration of the approved 2 g/day dose of the commercial Yomesan^®^ product, and to determine the extent to which once-daily dosing can achieve and maintain plasma concentrations within the range with reported in vitro antiviral activity. This information will be useful in guiding reformulation efforts to optimize systemic exposure of niclosamide for antiviral and other indications.

## 2. Materials and Methods

### 2.1. Study Design

This was a single-center, open-label, randomized, two-period crossover, multiple-dose, food-effect Phase 1 study. The study aimed to determine whether steady-state plasma niclosamide concentrations considered therapeutic for Zika virus treatment could be achieved using once-daily dosing of 2 g niclosamide, administered as the approved commercial formulation (Yomesan^®^ 500 mg tablets; Akesa Pharma, Melbourne, Australia).

**Figure 1:**
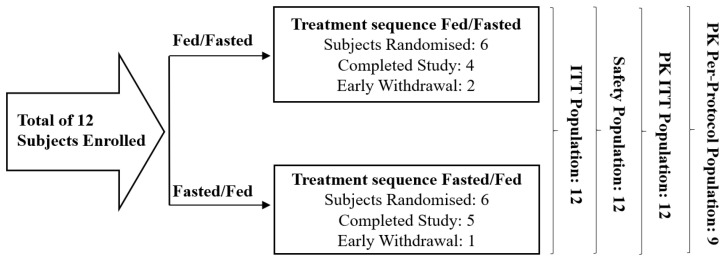
Study flow and analysis populations. Diagram showing subject enrollment, randomization to treatment sequence, study completion, and inclusion in intent-to-treat, safety, and pharmacokinetic analysis populations.

Twelve participants were randomized (1:1) to receive niclosamide initially under either fed or fasted conditions (Period 1), followed by crossover to the alternate condition after a 14-day washout (Period 2). Each dosing period consisted of three consecutive daily doses, with participants confined to the study unit for four nights per period (Days 1–4 and Days 17–21) (Figure 1). A follow-up telephone assessment was conducted seven days after completion of Period 2.

### 2.2. Study Population

The study was approved by the Australian Human Research Ethics Committee/The Alfred Hospital Ethics Committee (Local Reference No. 17/18). All participants provided written informed consent prior to study procedures.

Twelve healthy adults (six male, six female), aged 21–61 years, were enrolled. The study site opened for screening and related activities on 26 August 2018, and the first participants were enrolled on 23 September 2018, with the study commencing the following day. Eligibility required clinically insignificant findings on medical history, physical examination, vital signs, electrocardiogram(ECG), and laboratory assessments. Participants also had a negative urine drug screen and alcohol breath test prior to study entry and agreed to comply with all study procedures.

Key exclusion criteria included clinically significant cardiovascular, pulmonary, hepatic, renal, hematologic, gastrointestinal, endocrine, immunologic, neurologic, dermatologic, or psychiatric disease; acute viral illness within six weeks of screening; pregnancy or inadequate contraception; recent participation in another investigational study (within 30 days); recent blood donation (within 45 days); known hepatitis or HIV infection; or use of medications with potential drug–drug interactions with niclosamide.

### 2.3. Study Conduct and Dosing

Participants fasted for at least 10 h prior to dosing and for four hours post-dose on all dosing days. Niclosamide was administered orally at a dose of 2 g (four 500 mg tablets) with 240 mL of water on Days 1–3 (Period 1) and Days 18–20 (Period 2).

In the fed condition, participants consumed a standardized high-fat meal of ~800 calories, with at least 50% of the calories derived from fat, within 30 min prior to dosing. The meal consisted of fried eggs, bacon, buttered toast, hash brown potatoes, and whole milk. In the fasted condition, niclosamide was administered without a preceding meal. Participants crossed over to the alternate condition in Period 2.

### 2.4. Sample Collection and Bioanalysis

Serial blood samples were collected for pharmacokinetics (PK) and safety assessments. Blood samples for PK analysis were obtained pre-dose (within 1 h) and at 0.25, 0.5, 0.75, 1, 1.5, 2, 3, 4, 6, 8, 12, and 24 h post-dose on Days 1, 3, 18, and 20.

Plasma niclosamide concentrations were quantified using a validated LC–MS/MS method adapted from previously reported preclinical assays [20] and optimized for human plasma [21]. Key validation data is summarized in the Supporting Materials. The lower limit of quantification was 10 ng/mL.

In the method validation, apparent absolute recovery values exceeded 100% (140.8–166.4%). This reflects matrix-induced ion enhancement during electrospray ionization when post-extraction spiked samples are compared with neat standards, rather than true extraction efficiency. Ion enhancement of this magnitude has been reported for niclosamide in protein-rich matrices under electronspray ionization (ESI)-negative conditions and does not affect quantitative performance when accuracy, precision, and linearity criteria are met, as was the case for this assay. Method validation for the LC-MS/MS assay was performed according to established bioanalytical validation criteria; a summary of the validation parameters is provided in Supplementary Table S1.

No efficacy assessments were performed in this Phase 1 study.

### 2.5. Statistical Analysis

The study was descriptive in nature and not powered to detect statistically significant differences between treatment conditions. Safety and PK data were summarized using descriptive statistics only.

Quantitative variables were summarized using arithmetic mean, standard deviation (SD), median, minimum, and maximum values. Qualitative variables were summarized using frequency counts. Missing data were not imputed.

### 2.6. Analysis Populations

- Safety Population/Intention to Treat (ITT) Population: All randomized participants who received at least one dose of niclosamide.
- PK ITT Population: All participants who received any niclosamide dose.
- PK Per-Protocol (PK PP) Population: Participants who received niclosamide under both fed and fasted conditions and had sufficient PK samples to characterize PK parameters.

### 2.7. Safety Assessments

Adverse events (AEs) were coded using the Medical Dictionary for Regulatory Activities (MedDRA) [22]. Treatment-emergent adverse events (TEAEs) were summarized by treatment condition (fed vs. fasted), system organ class, preferred term, severity, and relationship to study drug. A subject-level summary of niclosamide adverse events and pharmacokinetic parameters is presented in Supplementary Table S2.

Clinical laboratory values, vital signs, and ECG parameters were summarized by treatment condition and time point, including changes from baseline and occurrences outside reference ranges. Physical examinations, concomitant medications coded using the World Health Organization (WHO) Drug Dictionary [23], and medical history were listed by participants.

### 2.8. Pharmacokinetic Analysis

Plasma PK parameters were derived using non-compartmental analysis and included area under the plasma concentration-time curve from time zero to the last quantifiable concentration (AUC_last_), area under the plasma concentration-time curve from time zero extrapolated to infinity (AUC_0–∞_), C_max_, time to maximum plasma concentration (T_max)_, terminal elimination rate constant (k_el_), elimination half-life (t½), apparent oral clearance (CL/F), and apparent volume of distribution (Vz/F). Parameters requiring a terminal log-linear phase were not reported when such a phase was absent.

Niclosamide concentration–time profiles were summarized and graphically presented for each treatment condition. Descriptive statistics included arithmetic and geometric means, SD, coefficient of variation, median, minimum, and maximum.

A food-effect analysis was performed using an Analysis of Variance (ANOVA) on log-transformed AUC_last_, AUC_0–∞_, and C_max_ values. Ratios of fed-to-fasted least squares means and corresponding 90% confidence intervals were calculated and expressed as percentages relative to the fasted condition.

### 2.9. Trial Organization

The study was conducted at the Centre for Clinical Studies in Melbourne, Australia, in collaboration with Clinical Network Services Pty Ltd. and Nucleus Network Limited, and in partnership with the College of Medicine, Florida State University, Tallahassee, FL, USA. This open-label, cross-over Phase I study was prospectively registered with the Australian New Zealand Clinical Trials Registry (ANZCTR) under registration number ACTRN12618001441202 on 28 August 2018.

## 3. Results

### 3.1. Study Population and Disposition

Twelve healthy adults (6 males, 6 females) were randomized to fed–fasted or fasted–fed treatment sequences (*n* = 6 per sequence). Only 11 of the randomized subjects participated in the study and comprised the ITT population. Of these, 9 participants (75%) completed both treatment conditions per protocol and comprised the per protocol population. For PK evaluations the ITT population was utilized. Because each subject needed both fed and fasted conditions, the per protocol population was used to assess the food effect. Failures to complete were for non–study-related reasons (two due to work or family commitments and one due to withdrawal of consent). No withdrawals were related to adverse events. No imputation of missing data was performed; pharmacokinetic analyses were based on observed data, with all available data from participants who discontinued early included up to the time of withdrawal. As missing data were limited to a single treatment arm in a small number of participants, a substantial impact on the comparison between fed and fasted conditions is unlikely based on the observed results, although a minor risk of bias cannot be excluded.

The mean age was 33.3 years (SD: 12.1; range: 21–61). Half of participants were female (*n* = 6). Most subjects were White (58.3%), with additional representation from Asian (25.0%), Black or African American (8.3%), and multiracial (8.3%) groups. Mean height, weight, and body mass index (BMI) were 172.3 cm (SD: 7.3), 70.6 kg (SD: 9.9), and 23.7 kg/m^2^ (SD: 2.7), respectively. All enrolled participants satisfied the eligibility criteria.

Thirty-one protocol deviations were recorded, primarily minor deviations from scheduled collection windows. None were deemed clinically meaningful or compromised safety, PK interpretation, or study integrity. The pharmacokinetic per-protocol (PK PP) population consisted of 9 subjects with evaluable PK data under both fed and fasted conditions.

### 3.2. Plasma Niclosamide Concentrations

**Figure 2:**
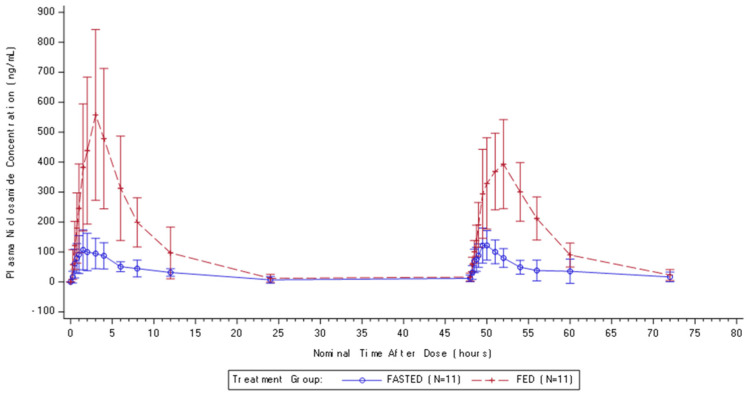
Mean Plasma Concentrations Over Time in nmol/L by Treatment (Linear) in PK ITT population.

Mean plasma niclosamide concentrations over time are summarized in Table 1 and illustrated in Figure 2. Across all post-dose sampling time points, plasma niclosamide concentrations were consistently and substantially higher under fed conditions compared with fasted conditions, following both initial and repeat dosing.

This pattern was observed for arithmetic mean, geometric mean, median, and maximum values. Inter-subject variability was generally lower under fed conditions, as reflected by consistently reduced coefficients of variation relative to the fasted condition.

Samples with concentrations below the lower limit of quantification (LOQ; 10 ng/mL) were more common under fasted conditions and occurred predominantly at early post-dose or trough time points. Fewer sub-LOQ samples were observed following fed administration.

Mean concentration–time profiles were qualitatively similar in shape between treatment conditions, but uniformly right-shifted and elevated in the fed state. Importantly, mean plasma concentrations under fed conditions exceeded the reported in vitro IC_50_ for Zika virus (0.22 µM) for approximately 9 h post-dose, whereas fasted administration did not consistently achieve this threshold.

### 3.3. Pharmacokinetics and Effect of Food

A pronounced food effect on niclosamide pharmacokinetics was demonstrated. On both Day 1 and Day 3, systemic exposure was substantially greater when niclosamide was administered with food, consistent with enhanced oral bioavailability.

Mean C_max_ was approximately 4.5-fold higher on Day 1 and 3.2-fold higher on Day 3 under fed conditions compared with fasted administration. Similarly, mean exposure parameters (AUC_last_, area under the plasma concentration–time curve over one dosing interval at steady state (AUCτ), and AUC_0–∞_) were consistently higher in the fed state. For every evaluable subject, C_max_ and AUC values were greater following fed administration.

As summarized in Table 2, mean C_max_ values in the fed condition were 607.3 ng/mL (CV 43.2%) on Day 1 and 459.6 ng/mL (CV 29.9%) on Day 3, compared with 126.8 ng/mL (CV 46.6%) and 142.8 ng/mL (CV 32.5%) in the fasted condition, respectively.

Median T_max_ values were similar across treatment conditions (2–4 h post-dose), indicating that food primarily affected the extent, rather than the rate, of absorption.

Mean apparent elimination half-life (t_½_), apparent oral clearance (CL/F), and apparent volume of distribution (Vz/F) were higher under fasted conditions. These differences are consistent with reduced bioavailability following Fasted administration, rather than true changes in systemic clearance or tissue distribution. Exposure within each treatment condition was similar between Day 1 and Day 3, indicating no unexpected accumulation during repeated once-daily dosing.

Formal food-effect analysis showed large and consistent increases in exposure under fed conditions. On Day 1, fed-to-fasted ratios (90% CI) of geometric means were 452.2% (305.5–669.4%) for C_max_, 417.5% (288.9–603.2%) for AUC_last_, and 290.2% (181.2–464.7%) for AUC_0–∞_. The corresponding ratios on Day 3 were 315.2% (262.8–378.1%), 344.8% (262.8–452.5%), and 244.6% (187.6–319.0%), respectively. Although the ratios were modestly lower on Day 3, the food effect remained robust across dosing days.

### 3.4. Safety and Tolerability

Niclosamide administered orally at 2 g daily for three consecutive days was well tolerated under both fed and fasted conditions. All treatment-emergent adverse events (TEAEs) were mild or moderate and transient. No severe, serious, or life-threatening TEAEs occurred, and no subject discontinued due to adverse events.

A total of 15 TEAEs were reported: 8 under fasted conditions and 7 under fed conditions, with no meaningful difference between treatments. The most reported TEAEs involved the nervous system and gastrointestinal system.

Gastrointestinal disorders accounted for the majority of treatment-related TEAEs. Under fed conditions, two subjects experienced three mild gastrointestinal events (abdominal discomfort, diarrhea); under fasted conditions, one subject reported mild nausea. Somnolence was considered treatment-related in two subjects (mild in fed, moderate in fasted), and one subject experienced a mild rash considered related to niclosamide.

No clinically significant abnormalities or trends were observed in clinical laboratory tests, urinalysis, vital signs, ECGs, or physical examinations under either dietary condition.

## 4. Discussion

Zika virus is a mosquito-borne flavivirus first identified in 1947 and associated with generally mild and self-limiting disease in most infected individuals [24]. However, Zika virus infection during pregnancy has been causally linked to severe congenital abnormalities, including microcephaly and other central nervous system malformations [25,26]. In addition, Zika virus infection has been associated with Guillain–Barré syndrome, a serious immune-mediated peripheral neuropathy [27]. There are currently no approved antiviral therapies or licensed vaccines for Zika virus, highlighting a significant unmet medical need.

Niclosamide is an orally administered antihelminthic drug approved for the treatment of intestinal tapeworm infections, including *Taenia saginata*, *Taenia solium*, *Diphyllobothrium latum*, and *Hymenolepis nana* [26]. In cell-based screening studies, niclosamide has demonstrated antiviral activity against Zika virus, inhibiting infection across multiple cell types at concentrations as low as approximately 0.02 µM and achieving complete inhibition at concentrations near 0.4 µM [19,28]. These in vitro potency values represent the only published quantitative benchmarks for niclosamide’s anti-Zika activity and therefore serve as reference points for contextualizing human plasma concentrations, although they do not by themselves establish clinical efficacy.

The currently marketed niclosamide formulation was developed for gastrointestinal infections and intentionally exhibits poor systemic bioavailability, limiting absorption beyond the gastrointestinal tract. In this Phase I study, we investigated the pharmacokinetics, safety, and tolerability of repeated oral administration of the approved immediate-release formulation at the maximum labeled daily dose of 2 g, under both fed and fasted conditions, to evaluate its potential to achieve plasma concentrations relevant to antiviral activity. Because this formulation was not designed to support systemic exposure, these data provide an initial pharmacokinetic foundation for future formulation optimization rather than a definitive assessment of antiviral therapeutic potential.

Under fed conditions, mean plasma niclosamide concentrations exceeded approximately 0.4 µM—the concentration associated with complete viral inhibition in vitro—from 45 min to approximately 8 h post-dose on both Day 1 and Day 3 and exceeded the IC_50_ for inhibition of Zika virus replication for approximately 9 h. At all other assessed post-dose time points, mean plasma concentrations remained above approximately 0.02 µM. In contrast, under fasted conditions, mean plasma niclosamide concentrations exceeded 0.02 µM at all post-dose time points but did not reach 0.4 µM at any time. These comparisons are intended solely to illustrate the relationship between observed plasma concentrations and published in vitro potency values; whether such exposures are therapeutically meaningful will require PK/PD characterization and clinical evaluation in Zika-infected individuals. A further limitation is that these comparisons rely on total plasma concentrations and do not account for niclosamide’s extensive protein binding (>99%). Because in vitro potency estimates reflect unbound drug, total plasma concentrations likely overestimate the pharmacologically active fraction in vivo. Accordingly, these values should be interpreted only as contextual reference points rather than indicators of expected antiviral efficacy.

Consistent with these observations, pharmacokinetic analysis demonstrated a pronounced food effect, with substantially higher systemic exposure following fed administration. C_max_ and AUC values were several-fold higher under fed conditions on both dosing days, with reduced inter-subject variability compared with fasted administration. Median T_max_ values were similar between treatment conditions, indicating that food primarily increased the extent of absorption rather than altering the rate of absorption. Apparent oral clearance and volume of distribution were higher under fasted conditions, consistent with reduced bioavailability rather than true changes in systemic drug disposition. Exposure within each treatment condition was consistent between Day 1 and Day 3, indicating no unexpected accumulation during repeated once-daily dosing.

Niclosamide was well tolerated under both fed and fasted conditions. All treatment-emergent adverse events were mild to moderate, transient, and consistent with the known safety profile of this approved drug. No serious or severe adverse events occurred, and there were no treatment-related withdrawals. In addition, no clinically meaningful changes were observed in laboratory parameters, vital signs, ECGs, or physical examinations. Although the open-label design may introduce reporting bias for subjective symptoms such as nausea or somnolence, the adverse events reported in this exploratory Phase 1 study were mild, transient, and in line with prior clinical experience. Given that the primary objective was pharmacokinetic characterization, the safety findings are descriptive and should be interpreted within the broader context of established tolerability data for niclosamide.

Taken together, these findings provide an initial characterization of niclosamide pharmacokinetics and short-term tolerability under fed and fasted conditions. However, several limitations should be considered when interpreting these results. The study enrolled a small cohort of healthy volunteers, and only nine participants completed the full crossover protocol, which limits the precision of the pharmacokinetic estimates. The short dosing duration further restricts conclusions regarding longer-term safety, tolerability, or drug accumulation. Because no efficacy or pharmacodynamic endpoints were included, and the population did not include Zika-infected individuals, pregnant participants, or other clinically relevant target groups, the clinical relevance of the exposure data remains exploratory. The fed condition employed a standardized high-fat meal that may not reflect typical dietary patterns during routine use. Interpretation of antiviral relevance is also constrained by reliance on total plasma niclosamide concentrations rather than unbound or tissue-level exposures at sites of infection, given the drug’s high protein binding. Finally, the small sample size limits the ability to detect uncommon adverse events, and the findings may be specific to the immediate-release formulation evaluated rather than directly generalizable to future optimized niclosamide formulations.

Collectively, these results demonstrate that administration of the approved immediate-release niclosamide formulation with food can achieve transient systemic plasma concentrations that exceed published in vitro antiviral potency thresholds for Zika virus for several hours following dosing. While sustained 24 h exposure at these levels was not achieved with the current formulation, these findings provide a pharmacokinetic benchmark to guide the development of optimized niclosamide formulations. Future efforts aimed at improving systemic bioavailability—such as enhanced formulations, divided dosing strategies, and/or modified-release approaches—may enable more sustained plasma concentrations capable of supporting formal PK/PD modeling and, ultimately, clinical evaluation in Zika-infected individuals.

## 5. Conclusions

This Phase I study demonstrated that repeated once-daily oral administration of niclosamide at 2 g was safe and well tolerated in healthy volunteers under both fed and fasted conditions. Niclosamide exposure was markedly higher when administered with food, with plasma concentrations transiently exceeding published in vitro potency values associated with complete inhibition of Zika virus infection for approximately 9 h post-dose.

These data support further clinical investigation of niclosamide formulations optimized to achieve more sustained systemic exposure, including plasma concentrations exceeding approximately 0.2 µM over a 24 h period, while maintaining the approved daily dose limit of 2 g. Such studies will be necessary to determine whether these exposure targets are clinically meaningful.

## 6. Patents

Holmes, E.; Ostrander, G. Niclosamide formulations and methods of use. U.S. Patent 11,771,668 B2, issued 3 October 2023; assigned to Florida State University Research Foundation, Inc.
Holmes, E.; Ostrander, G. Niclosamide formulations and methods of use. U.S. Patent 12,161,611 B2, issued 10 December 2024; assigned to Florida State University Research Foundation, Inc.
Holmes, E.; Ostrander, G. Niclosamide formulations and methods of use. U.S. Patent 12,427,126 B2, issued 30 September 2025; assigned to Florida State University Research Foundation, Inc.

## Figures and Tables

**Table 1:** Plasma Niclosamide Concentration in ng/mL (PK ITT population).

		**Plasma Concentration (nmol/L)**												
		**Scheduled Time Point**												
**Treatment**	**Statistics**	**Day 1,**	**Day 1,**	**Day 1,**	**Day 1,**	**Day 1,**	**Day 1,**	**Day 1,**	**Day 1,**	**Day 1,**	**Day 1,**	**Day 1,**	**Day 1,**	**Day 2**
		**Pre-Dose**	**15 min**	**30 min**	**45 min**	**1 h**	**1.5 h**	**2 h**	**3 h**	**4 h**	**6 h**	**8 h**	**12 h**	**24 h**
FED	N	11	11	11	11	11	11	11	11	11	11	11	11	11
	Mean	0	178.3	373.1	550.6	750.7	1169	1341	1705	1463	956.3	608.9	296.9	37.4
	SD	0	151.9	245.9	358.4	453.76	648.9	751	870.8	715.76	531.6	251.3	264.2	39.3
	CV (%)	0	85.2	65.9	65.1	60.4	55.5	56	51.1	48.9	55.6	41.3	89	105.3
	Median	0	161.7	360.7	541.1	871.2	1137	1275	1770	1394	816.2	638.9	214	42.8
	Minimum	0	0	0	43.4	75.5	193	266	694	474	291	306	0	0
	Maximum	0	550	883	1360	1720	2360	2510	3120	2860	2130	1020	939	133
	Geometric Mean	0	136.7	282.9	417.1	581.8	965.9	1116	1496	1297	832.5	561.4	208.7	43.8
	n BLQ	11	2	1	0	0	0	0	0	0	0	0	1	4
FASTED	N	11	11	11	11	11	11	11	11	11	10	10	10	10
	Mean	0	49.13	187.8	241.6	279	327	305.5	290.3	267.2	156.1	136.8	96.8	22.3
	SD	0	59.5	145.8	149.2	194.01	202.8	189.8	154.6	134.03	50.4	85.7	38.9	33.9
	CV (%)	0	121.1	77.7	61.8	69.5	62	62.1	53.3	50.2	32.3	62.6	40.2	151.9
	Median	0	45.9	177.3	251	269.6	304.5	272.7	327.1	273	164.5	129.2	95.2	0
	Minimum	0	0	60.5	50.4	53.8	77	69.7	68.5	113	59.6	57.2	47.7	0
	Maximum	0	207	569	593	764	828	746	578	517	245	355	153	102
	Geometric Mean	0	50.2	149.7	200.3	224.4	275.3	253.1	246.8	235.7	147.1	119	89.9	37.8
	n BLQ	11	4	0	0	0	0	0	0	0	0	0	0	6
		**Plasma Concentration (nmol/L)**												
		**Scheduled Time Point**												
**Treatment**	**Statistics**	**Day 3,**	**Day 3,**	**Day 3,**	**Day 3,**	**Day 3,**	**Day 3,**	**Day 3,**	**Day 3,**	**Day 3,**	**Day 3,**	**Day 3,**	**Day 3,**	**Day 4,**
		**Pre-Dose**	**15 min**	**30 min**	**45 min**	**1 h**	**1.5 h**	**2 h**	**3 h**	**4 h**	**6 h**	**8 h**	**12 h**	**24 h**
FED	N	11	11	11	11	11	11	11	11	11	11	11	11	11
	Mean	49.94	178.6	308.2	421.7	583.4	899	1006	1127	1203	919.3	647.8	275.2	70.67
	SD	41.14	74.26	115.35	158.24	228.14	454.38	465.67	391.69	453.9	299.15	219.97	123.31	55.39
	CV (%)	82.4	41.6	37.4	37.5	39.1	50.5	46.3	34.7	37.7	32.5	34	44.8	78.4
	Median	58.08	185.6	339.3	458.6	638.9	865.1	950.7	1183	1241	822.3	629.7	273.6	70.62
	Minimum	0	41.9	99.4	131	227	333	413	492	535	480	315	48.3	0
	Maximum	138	324	511	676	853	1940	2010	1710	2050	1530	1150	477	162
	Geometric Mean	52.4	160.8	283.9	386.1	533.9	802.1	909.6	1056	1120	876.6	615	240.6	67.5
	n BLQ	3	0	0	0	0	0	0	0	0	0	0	0	3
FASTED	N	10	9	9	9	9	9	9	9	9	9	9	9	9
	Mean	36.8	97.11	213.9	230.6	269.8	371.3	373.3	307	244.6	150.6	117.1	109	51.6
	SD	35.16	70.34	119.66	122.23	120.08	178.19	150.03	120.69	95.2	70.43	105.52	124.22	49.09
	CV (%)	95.4	72.4	55.9	53	44.5	48	40.2	39.3	38.9	46.8	90.1	114	95.1
	Median	43.72	74.59	185.9	195.3	214	382.1	400.5	321	225.3	161.7	102.7	60.83	40.66
	Minimum	0	0	88	84.7	129	153	134	110	88	78.6	41.6	30.6	0
	Maximum	90.5	241	397	407	434	651	544	468	440	312	385	428	138
	Geometric Mean	45.51	84.47	185.1	200.2	245.4	330.5	337	281	226.9	138.5	91.98	76.77	53.2
	n BLQ	4	1	0	0	0	0	0	0	0	0	0	0	3

Notes: For the calculation of summary statistics BLQ values were set to zero, except for geometric mean where BLQ values were set to the lower limit of quantification. Lower limit of quantification is 31 nmol/L. BLQ = Below Limit of Quantification. SD = Standard Deviation; CV = Coefficient of Variation. Treatments: FED = Niclosamide 2 g/day in fed state; FASTED = Niclosamide 2 g/day in fasted state.

**Table 2:** Plasma Niclosamide Concentration in nmol/L (PK ITT population).

			**Pharmacokinetic Parameters**								
**Treatment**	**Visit**	**Statistic**	**Cmax**	**Tmax**	**AUC(0-last)**	**AUCtau**	**AUC(0-inf)**	**K_el_**	**t1/2**	**CL/F**	**Vz/F**
			**(ng/mL)**	**(h)**	(h*ng/mL)	(h*ng/mL)	(h*ng/mL)	**(1/h)**	**(h)**	**(L/h)**	**(L)**
FED	Day 1	N	11	11	11	10	10	10	10	10	10
		Mean	607.3	3.1	3941	4188	4273	0.2	3.8	554.5	2960
		SD	262.4	1.8	1870.8	1821	1874	0.1	1.4	237.2	1514.4
		CV (%)	43.2	58.2	47.5	43.5	43.9	40.4	37.5	42.8	51.2
		Median	586	3	3141	3723	3678	0.2	3.8	550.1	2691
		Minimum	276	1.5	1640	2000	2000	0.1	1.8	253	1280
		Maximum	1020	8	7810	7820	7900	0.4	6.6	999	6110
		Geometric Mean	552.8	NA	3569	3851	3923	0.2	NA	509.9	2636
		Geometric CV	49.3	NA	49.4	45.4	45.9	40.3	NA	45.9	54.7
	Day 3	N	11	11	11	10	9	9	9	9	9
		Mean	459.6	3.8	3512	3657	3964	0.2	5.1	573.1	3963
		SD	137.3	1.9	1084.9	1038.4	1217.2	0.1	2.2	152.9	1591.8
		CV (%)	29.9	49.9	30.9	28.4	30.7	69.5	42.9	26.7	40.2
		Median	430	4	3041	3171	3268	0.1	4.9	625.1	4354
		Minimum	273	1.5	2230	2550	2550	0.1	1.5	357	1740
		Maximum	671	8	5610	5610	5890	0.5	8.2	785	6350
		Geometric Mean	441.3	NA	3371	3534	3803	0.2	NA	554.1	3633
		Geometric CV	30.8	NA	30.2	27.7	31.1	60.7	NA	28.5	49.1
			**Pharmacokinetic Parameters**								
**Treatment**	**Visit**	**Statistic**	**Cmax**	**Tmax**	**AUC(0-last)**	**AUCtau**	**AUC(0-inf)**	**K_el_**	**t1/2**	**CL/F**	**Vz/F**
			**(ng/mL)**	**(h)**	(h*ng/mL)	(h*ng/mL)	(h*ng/mL)	**(1/h)**	**(h)**	**(L/h)**	**(L)**
FASTED	Day 1	N	11	11	11	9	7	7	7	7	7
		Mean	126.8	3.045	823	1009	1114	0.1	5.9	2011	16,620
		SD	59.1	1.4	395.3	314.1	364.1	0.04	1.9	797.9	6314.2
		CV (%)	46.6	46.1	48	31.1	32.7	31.6	32	39.7	38
		Median	118	3	817.5	1130	1288	0.1	5.3	1552	17,210
		Minimum	55.4	1.5	182	551	573	0.1	3.6	1290	9100
		Maximum	271	6	1450	1460	1550	0.2	9	3490	26,000
		Geometric Mean	115.5	NA	714.3	962.9	1056	0.1	NA	1893	15,540
		Geometric CV	48.1	NA	67.8	34.2	37.9	32.8	NA	37.9	42
	Day 3	N	9	9	9	9	5	5	5	5	5
		Mean	142.8	3.1	1016	1046	919.7	0.1	7.8	2907	29,620
		SD	46.4	3.3	492.8	474.8	394.9	0.1	4.6	1426.4	16,024
		CV (%)	32.5	107.7	48.5	45.4	42.9	63.6	59.5	49.1	54.1
		Median	146	2	1028	1066	933.4	0.1	5.539	2815	33,030
		Minimum	51.8	1.5	317	378	391	0.1	2.8	1720	11,600
		Maximum	213	11.8	2090	2090	1420	0.2	13.3	5280	49,200
		Geometric Mean	134.1	NA	910.1	953.1	841.2	0.1	NA	2672	25,650
		Geometric CV	42.8	NA	55.6	49.8	53.1	72	NA	46.8	69.8

Notes: Treatments: FED = Niclosamide 2 g/day in fed state. FASTED = Niclosamide 2 g/day in fasted state. NA = Not Applicable.

## Data Availability

De-identified participant data are available from the corresponding authors upon reasonable request, subject to institutional and ethical approval.

## Supplementary Materials

The following supporting information can be downloaded at: https://www.mdpi.com/article/10.3390/jcm15145330/s1, Table S1: Validation Summary for the LC-MS/MS Analysis; Table S2: Summary of individual niclosamide Adverse Events and PK parameters. AEs only occurred during Period 1 and blank cells indicate individuals who declined further participation.

## Abbreviations

The following abbreviations are used in this manuscript:

AEs	Adverse events
AUC	Area under the drug concentration-time curve
AUCtau	Area under the concentration–time curve over one dosing interval
AUC(0–∞)	Area Under the Curve from time zero to infinity
AUC(0-last)	Area Under the Curve to the last measurable concentration
AUC(0-t)	Area Under the Curve from time zero to time t
BMI	Body Mass Index
CI	Confidence Interval
CL/F	Apparent Oral Clearance after single dose
Cmax	Maximum observed plasma concentration after dosing
CV	Coefficient of Variation
ECG	Electrocardiogram
FDA	Food and Drug Administration
IC_50_	Half maximal inhibitory concentration
ITT	Intention-to-Treat
LC/MS/MS	Liquid Chromatography/Tandem Mass Spectrometry
LD50	Median lethal dose
LOQ	Limit of Quantification
MedDRA	Medical Dictionary for Regulatory Activities
n BLQ	Number of samples below the limit of quantitation
PK	Pharmacokinetics
PP	Per-Protocol
SAEs	Serious Adverse events
SD	Standard deviation
TEAEs	Treatment-Emergent Adverse Events
T_max_	Time to Maximum Concentration
T1/2	Half-life
Vz/F	Apparent Volume of Distribution during the Terminal Phase After Dosing
WHO	World Health Organization
