# Supplementary material for: An Open Label, Cross-Over Phase 1 Study to Determine the Safety, Tolerability and Pharmacokinetics of Multiple Oral Doses of Niclosamide Under Fed and Fasted Conditions in Healthy Volunteers
Source: J Clin Med. 2026 Jul 8;15(14):5330. doi: 10.3390/jcm15145330 (PMC13411445; doi:10.3390/jcm15145330)
Supplement: Supplementary file 1 [file jcm-15-05330-s001.zip › jcm-4282403-supplementary.pdf]

Table S1. Validation Summary for the LC-MS/MS Analysis.

Matix (anticoagulant)	Human Plasma (K_2EDTA)
Internal Standard	$^{13}C_6$-Niclosamide hydrate (IS)
Technique	Protein precipitations then LC-MS/MS
Calibration Range	10-10,000 ng/ml
Selectivity	No endogenous interferences observed<br>Matrix Effects: No Significant Matrix Effects observed<br>Hemolysis evaluation: No significant effects on quantification<br>Lipemic evaluation: No significant effects on quantification<br>OTC drugs: no interference with the analyte was observed when tested with Acetaminophen, Amoxicillin, Aspirin, Caffeine, Chlorpheniramine Maleate, Desipramine, Ibuprofen, Lidocaine, Naproxen, Pseudoephedrine, Salicylic Acid, Theobromine, theophylline, Tetracycline, and Xanthine.<br>IS Normalized Matrix Factor – 1.02 (QC Low – 30.0 ng/ml)<br>IS Normalized Matrix Factor – 0.986 (QC High – 7500 ng/ml)
Linearity	Coefficient of determination for curves run during validation $r^2 \geq 0.9943$
Regression used	Linear $1/x^2$ weighting
Inter-Assay Precision (Mean % CV)	Between -3.1 and 3.0<br>N=18 (n=17 for PQCL)
Inter-Assay Precision (% Bias)	Between 4.8 and 6.5<br>N=18 (n=17 for PQCL)
Inter-Assay Precision (Mean % CV)	Between 1.9 and 8.4; N=6 x 3 (n=6 x 2 on two occasions and 5 x 1 on one occasion for PQCL)
Inter-Assay Precision (% Bias)	Between -7.7 and 4.8; N=6 x 3 (n=6 x 2 on two occasions and 5 x 1 on one occasion for PQCL)
Analyte Recovery	166.4% GC Low (30.0 ng/ml); CV = 6.7%<br>154.7% QC Mid (750 ng/ml); CV = 6.0%<br>140.8% QC High (7500 ng/ml); CV = 1.4%
IS Recovery	151.9% (25.0 ng/ml); CV = 10.6%
Stabilities	Freeze–thaw cycle (nominal -20 C and -80 C)<br>Short term stability at RT: 24 hours<br>Re-injection reproducibility at RT: 136.5 hours<br>Extracted sample stability at RT: 99.25 hours<br>Whole Blood stability on ice and RT: 1 hour<br>Analyte stock solution (25% DMF 75% ACN) stability when stored @ 4 C: 62 days<br>Analyte stock solution (25% DMF 75% ACN) stability when stored @ RT: 25 hours<br>IS stock solution (analyte stabilities apply)<br>Long Term Stability in plasma at -80 C: 66 days

	Long Term Stability in plasma at -20 C: 66 days

Table S2. Summary of individual niclosamide Adverse Events and PK parameters. AEs only occurred during Period 1 and blank cells indicate individuals who declined further participation.

RND	Dose Group	AEs by preferred term	SAE?	Cmax Fed Day 1	Cmax Fasted Day 1	Cmax Fed Day 3	Cmax Fasted Day 3
1011	Fasted	Headache	No	901	55.4	559	152
1010	Fasted	Facial Rash	No	1020	66.7	585	
1008	Fed	Drowsiness	No	417		656	
1008	Fed	Abdominal Discomfort	No	417		656	
1008	Fed	Upper Respiratory Tract Infection	No	417		656	
1107	Fed	Lethargy	No	276	341	107	113
1107	Fed	Gastrointestinal Upset	No	276	341	107	113
1005	fasted	Drowsiness	No		104		BLQ
1005	Fasted	Nausea	No		104		BLQ
1005	Fasted	Upper Respiratory Tract Infection	No		104		BLQ
